# Longitudinal changes in cortical responses to letter-speech sound stimuli in 8–11 year-old children

**DOI:** 10.1038/s41539-021-00118-3

**Published:** 2022-01-25

**Authors:** Linda Romanovska, Roef Janssen, Milene Bonte

**Affiliations:** Maastricht Brain Imaging Center, Department Cognitive Neuroscience, Faculty of Psychology and Neuroscience, Maastricht University, Maastricht, The Netherlands

**Keywords:** Education, Scientific community

## Abstract

While children are able to name letters fairly quickly, the automatisation of letter-speech sound mappings continues over the first years of reading development. In the current longitudinal fMRI study, we explored developmental changes in cortical responses to letters and speech sounds across 3 yearly measurements in a sample of 18 8–11 year old children. We employed a text-based recalibration paradigm in which combined exposure to text and ambiguous speech sounds shifts participants’ later perception of the ambiguous sounds towards the text. Our results showed that activity of the left superior temporal and lateral inferior precentral gyri followed a non-linear developmental pattern across the measurement sessions. This pattern is reminiscent of previously reported inverted-u-shape developmental trajectories in children’s visual cortical responses to text. Our findings suggest that the processing of letters and speech sounds involves non-linear changes in the brain’s spoken language network possibly related to progressive automatisation of reading skills.

## Introduction

Reading is an evolutionary novel, culturally acquired skill that requires us to associate speech with visual symbols. The initial mappings between letters and speech sounds are typically acquired within the first months of reading instruction^1,2^, particularly in orthographically transparent languages with relatively consistent letter-speech sound associations such as Dutch, German, Italian or Hungarian^3^. However, the fine-tuning and automatisation of letter-speech sound integration continues while children strengthen their word decoding skills over the first years of reading development^1,4–6^.

During this protracted developmental period, brain areas involved in speech and visual letter processing become increasingly connected both functionally and structurally^7–11^. This drives the adaptation of the individual brain areas for the task of reading. For example, a region in the (left) higher order visual cortex, often referred to as the visual word form area (VWFA), becomes increasingly specialised for text processing^12–15^. The continuous functional specialisation within this brain region has been proposed to follow an inverted-u-trajectory, showing an increase in activation during early reading instruction followed by a gradual decrease in activation with reading expertise^16^. This pattern of cortical activation in the VWFA has been reported during the first year of formal reading instruction in response to visual word and letter presentation^15,17^, as well as when comparing cortical responses to text longitudinally between kindergarten and second grade vs. adult readers^14^. A similar non-linear pattern of developmental change can also be inferred from across study comparisons of neural sensitivity to (in)congruency of letter-speech sound pairs^4,18,19^. Next to inverted-u-shape changes, other types of developmental trajectories, including linear changes, have also been observed for e.g. functional responses to speech sounds^20^, white matter connectivity^9^, and cortical grey- and white matter anatomy^21–25^.

The link between speech and text processing has typically been illustrated by modulations in cortical activation in response to matching (congruent) vs. nonmatching (incongruent) audio-visual letter-speech sound stimuli. Functional magnetic resonance imaging (fMRI) studies employing such congruency manipulations have shown increased activation of speech sensitive areas within the superior temporal cortex (STC) in response to congruent letter-speech sound pairs compared to incongruent pairs and speech alone^26–28^. Furthermore, the strength of STC responses to congruent audio-visual stimuli has been found to scale with individual differences in phonological and reading skills^29,30^ and with reading problems in dyslexic readers^31–34^. However, congruency manipulation paradigms rely on learnt letter-speech sound mappings and involve additional cognitive and verbal working memory processes that may vary with participants’ reading^35^ and selective attention^36^ skills.

An alternative approach to investigating letter-speech sound mappings can be found in learning paradigms, such as recalibration. Recalibration studies explore short-term perceptual learning associated with mapping ambiguous speech onto a disambiguating visual stimulus^37–41^. Here we employed text-based recalibration in a longitudinal fMRI design to follow changes in letter-speech sound processing in 8–11 year-old children. Our recalibration paradigm combines “aba” and “ada” text with an ambiguous /a?a/ speech sound mid-way between /aba/ and /ada/^41–44^. The clear text stimuli are combined with the ambiguous speech sound during audio-visual exposure blocks and shift the listener’s subsequent perception of the ambiguous /a?a/ sound towards the text during auditory-only post-test trials. Thus, after being exposed to “aba” text combined with the /a?a/ sound, the listener is more likely to perceive the ambiguous sound as /aba/, and after combining the same ambiguous speech sound with “ada” text, it is more likely to be perceived as /ada/. This shift in perception is called recalibration and has previously been reported using lip-read speech^37,38,40^, spoken word context^39,40^ and overt speech articulation^45^. Behaviourally, reliable text-based recalibration effects have been observed in 8–10 year old children^44^, where the strength of the recalibration effect was found to be associated with children’s categorical perception of phonemes. Functional MRI responses in typically reading adults have shown that the text-induced perceptual shifts can be decoded from activity patterns in the posterior STC and further involve a network of brain areas associated with audio-visual integration and reading, including visual, parietal and frontal regions^42^. A similar cortical network emerged during text-based recalibration in 8–10 year old children with and without dyslexia^46^.

Here we employed the text-based recalibration paradigm to investigate longitudinal developmental changes in the posterior STC and the broader audio-visual reading network in 8–11 year old children who were scanned yearly over a 3 year period. This study is part of a larger longitudinal fMRI project investigating letter-speech sound mapping in children with and without developmental dyslexia. We have previously reported group differences in brain activation during the audio-visual exposure blocks of the text-based recalibration task in the first fMRI session. Namely, despite comparable behavioural recalibration performance, children with and without dyslexia showed differences in ventral occipito-temporal cortex activation^46^. Here we investigated longitudinal changes in this audio-visual activation across the three sessions in a group of children without dyslexia. During the audio-visual exposure blocks, the children were simultaneously presented with the ambiguous /a?a/ sound and disambiguating text. Children’s responses during the subsequent auditory-only post-test trials were used to assess the magnitude of the recalibration effect behaviourally. Our text-based recalibration paradigm was specifically designed to investigate reading-induced changes in the left posterior STC^42^. We thus expected to find longitudinal changes in auditory cortical activation. To also investigate possible changes across other regions of the brain’s audio-visual reading network (see e.g.^46^), we did not restrict our analyses to the auditory cortex but performed whole brain analyses. In these analyses, we were particularly interested in possible links between children’s cortical responses to audio-visual letter-speech sound stimuli, their recalibration task performance, and individual differences in phonological perception and reading skills.

## Results

### Pre-test results

**Fig. 1:**
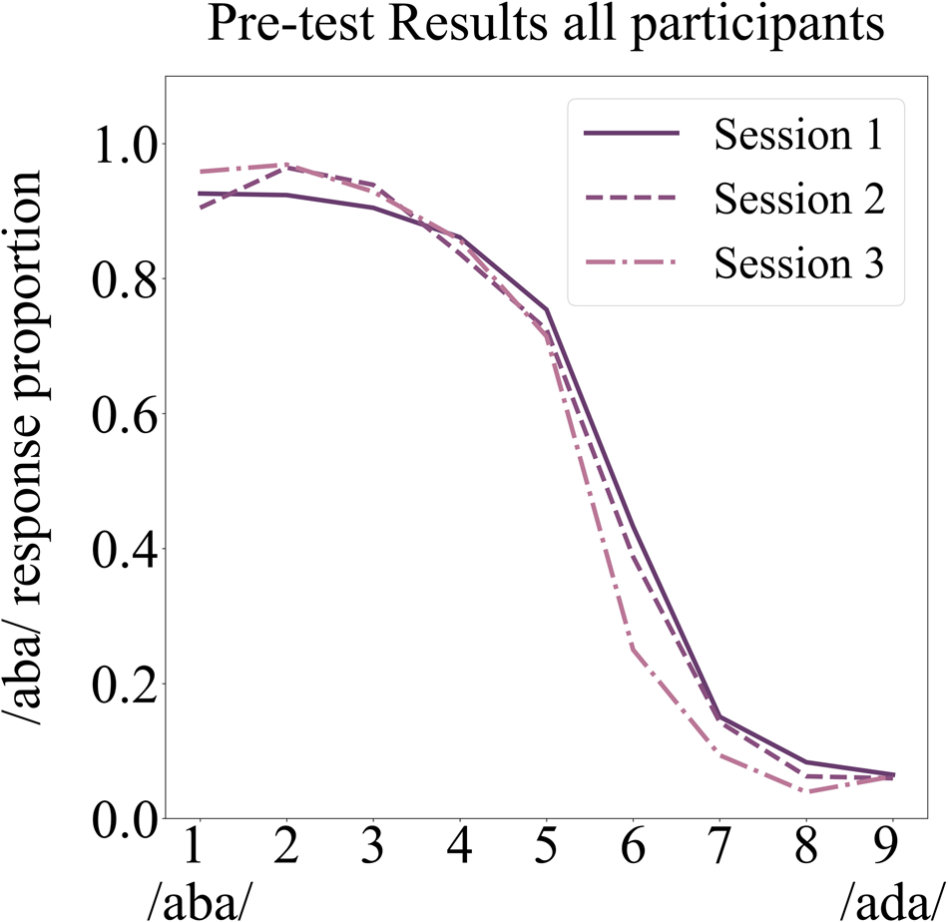
Pre-test results. The proportion of /aba/ responses to each of the 9 sound tokens (1–9) on the /aba/-/ada/ continuum across participants is shown for Session 1 (solid line); Session 2 (dashed line) and Session 3 (dotted dashed line).

Prior to the MRI, the children performed a pre-test during a mock scanner session (see Methods section) in order to determine the individual, most ambiguous sound (/a?a/) to be used during the text-based recalibration task. During the pre-test, the children heard nine sounds tokens along an /aba/–/ada/ continuum and for each sound indicated whether they perceived it as /aba/ or /ada/. The overall /aba/ response proportions to each of the nine sound tokens indicate that all participants perceived the sound tokens closer to the /aba/ end of the continuum as /aba/ and the sounds closer to the /ada/ end of the continuum as /ada/ (see /aba/ response proportions for tokens 1–3 and 7–9, Fig. 1). This was confirmed in a 3 (session) × 9 (sound) RM ANCOVA analyses of the /aba/ response proportions to each of the nine sounds, which revealed a main effect of sound [F(2,24) = 4.087, *p* < 0.05, Greenhouse-Geisser corrected] and an interaction between sound and children’s age in months during session 1 (baseline age) [F(2,24) = 5.629, *p* < 0.01, Greenhouse-Geisser corrected]. No other main- or interaction effects reached statistical significance (all *F* ≤ 2.97). This indicates that the average /aba/ response proportions to each of the nine sounds along the /aba/-/ada/ continuum did not significantly change across the three measurement sessions but that younger children on average showed a different /aba/ response proportion bias. Namely, their responses were biased more towards /aba/.

### Behavioural results recalibration task

**Fig. 2:**
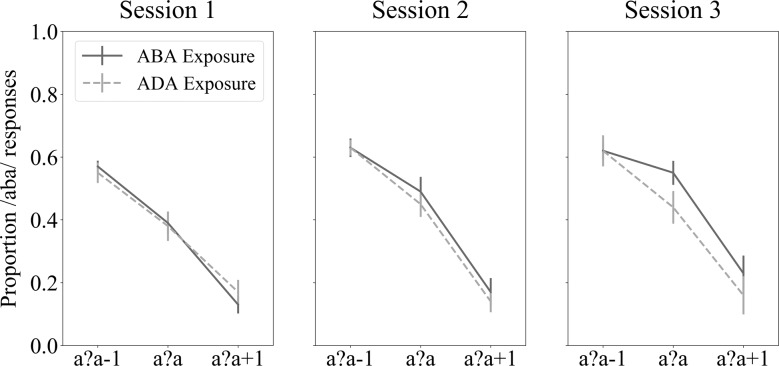
Behavioural text-based recalibration results. Each panel shows the /aba/ response proportions (*y*-axis) for each of the three post-test sound tokens (x-axis) following “aba” (solid) and “ada” (dashed) exposure blocks in each MRI session. Vertical bars indicate standard error of the mean.

Children’s responses to the auditory-only post-test sounds showed a change in /aba/ response proportions across the ambiguous sound and its two flanking sounds along the /aba/-/ada/ continuum (Fig. 2). There were more /aba/ responses for the sound /a?a/-1 closer to the clear /aba/ token, as well as for the ambiguous /a?a/ sound, compared to the /a?a/+1 sound that is closer to the clear /ada/ token. However, a 3 (session) × 2 (“aba” / “ada” exposure block) × 3 (post-test sounds) RM ANCOVA did not show significant main- or interaction effects (all *F* ≤ 2.19), indicating a lack of a significant behavioural recalibration effect across participants.

### fMRI activity during the exposure blocks

**Fig. 3:**
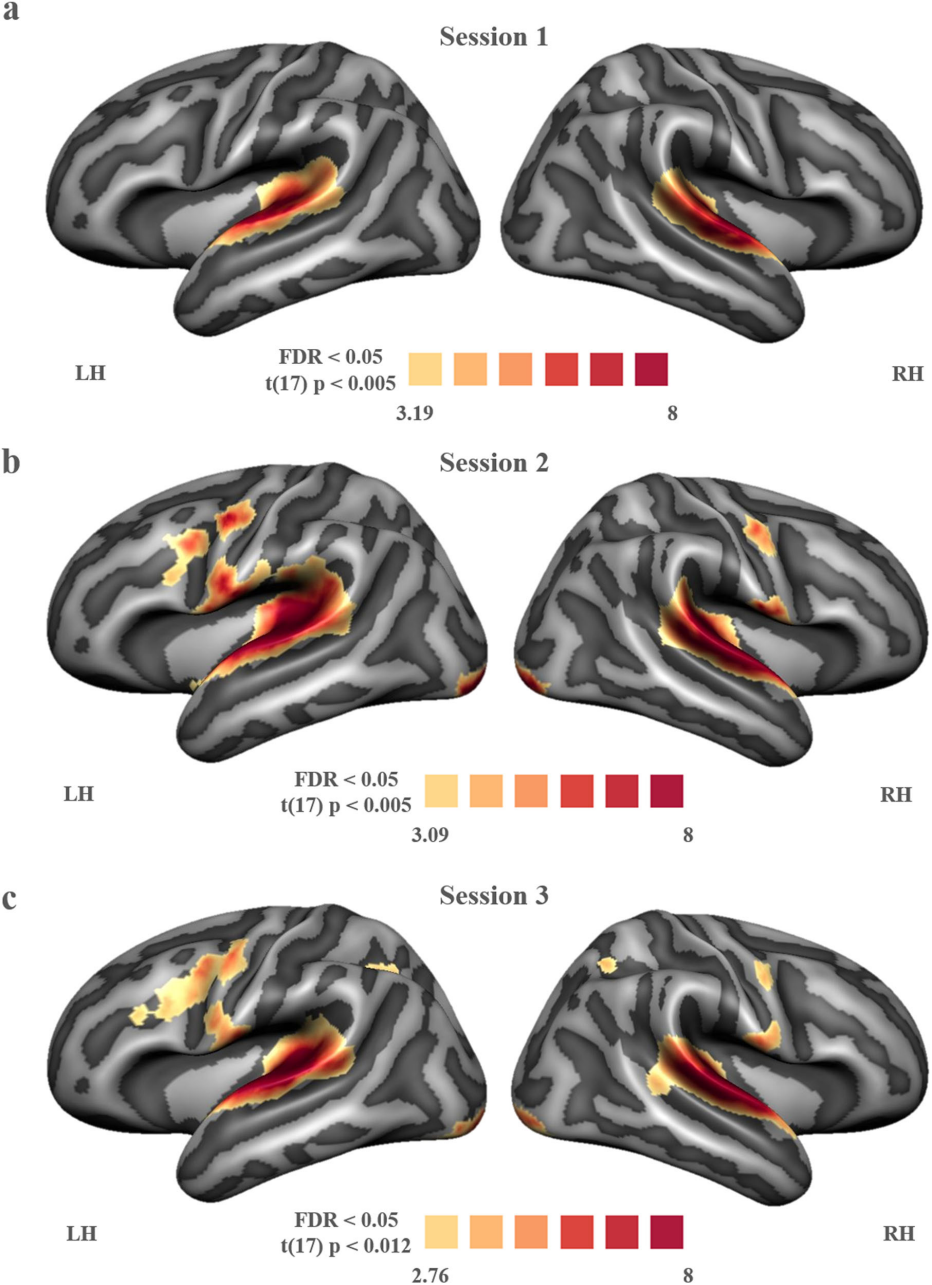
Cortical activation across all participants during the audio-visual exposure blocks compared to baseline. (**a**) measurement 1 (**b**) measurement 2 (**c**) measurement 3. LH = left hemisphere, RH = right hemisphere.

**Fig. 4:**
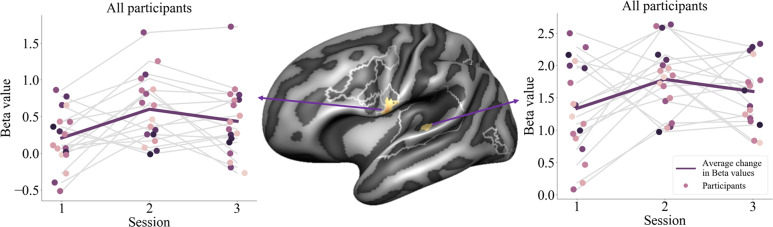
Left hemisphere STG and lateral inferior precentral gyrus clusters showing differences in cortical activation across sessions. Left (pre-CG) and Right (STG) individual beta values (*y*-axis) per participant (scatter dots) per session (*x*-axis). Grey lines represent the individual trajectories of change in cortical activation across sessions. White outlines represent the cortical mask used to restrict the RM ANOVA results.

The text and speech sound stimuli presented during exposure blocks of the recalibration task elicited significant activation in bilateral superior temporal gyri (STG), visual and parietal cortices, as well as in the left frontal cortex (Fig. 3; whole-brain FDR threshold *q* < 0.05). Talairach coordinates of activation clusters for each individual measurement session are reported in Table 1. At the given (FDR) threshold, the resulting maps appear to indicate a general broadening of this network from sessions 1 to 3. However, RM ANOVA analyses investigating changes in cortical activation across sessions, revealed significant clusters only in the left STG and lateral inferior precentral gyrus (pre-CG; Fig. 4; whole-brain FDR threshold *q* < 0.05). Plotting the average individual beta values per participant per session for visualisation purposes, indicates non-linear changes in cortical activation across sessions in both clusters (Fig. 4). Using a stringent correction for multiple comparisons (FDR correction using the Benjamini and Hochberg procedure), pairwise session comparisons only yielded a significant difference between sessions 1 and 2 in the lateral inferior pre-CG cluster (*t*(17) = −3.038, *p* < 0.01; *q* = 0.04), while at uncorrected level, activation in this cluster also differed between sessions 1 and 3 (*t*(17) = −2.571, *p* < 0.05; *q* = 0.06) and sessions 1 and 2 in the left STG cluster (*t*(17) = −2.211, *p* < 0.05; *q* = 0.08).

**Table 1:** Talairach coordinates of cortical activation clusters during the audio-visual exposure blocks compared to baseline for each measurement session.

Area	Hemisphere	Cluster size (n vertices)	Talairach coordinates (center of gravity)		
			*x*	*y*	*z*
Session 1					
STG	Left	1924	−58	−31	13
STG/STS	Right	1814	65	−23	15
Session 2					
STG/lateral M1	Left	3558	−53	−29	14
Frontal	Left	165	−49	7	41
M1	Left	217	−55	−11	55
V1	Left	161	−34	−106	−9
STG/STS	Right	2298	62	−23	15
Lateral M1	Right	291	65	−3	23
M1	Right	170	55	−7	55
V1	Right	144	35	−102	−4
Session 3					
STG	Left	2185	−59	−30	13
Frontal/M1	Left	525	−51	1	46
M1	Left	331	−69	−7	25
IPL	Left	270	−39	−70	48
V1	Left	166	−34	−105	−10
STG/STS	Right	2021	66	−23	15
Lateral M1	Right	180	69	−6	27
M1	Right	91	56	−5	54
IPL	Right	76	38	−67	58
V1	Right	134	36	−100	−6

*STG* superior temporal gyrus, *STS* superior temporal sulcus, *M1* primary motor cortex, *V1* primary visual cortex, *IPL* inferior parietal lobe, *pre-CG* precentral gyrus.

### Mixed regression results

To explore how children’s age, reading fluency and phoneme perception (pre-test slope steepness) may have influenced the change in left STG and lateral inferior pre-CG activity across sessions, we further assessed the individual beta values for each session with post-hoc mixed regression analyses. The analyses showed significant interactions between session and children’s baseline age [*F*(2,13) = 8.702, *p* < *0.005*] and between session and the recalibration effect [*F*(2,17) = 5.808, *p* < *0.05*] in the left STG cluster. Estimated marginal means comparisons showed that younger children showed higher left STG activation during session 2 compared to session 3 (mean difference = 0.596, *t*(11) = 2.950, *p* < *0.05*), whereas older children showed more STG activation in session 3 compared to session 2 (mean difference = 0.502, *t*(10) = 2.918, *p* < *0.05*). These analyses also revealed that, regardless of age, children who show a (stronger) recalibration effect across measurement sessions, show more activation in the left STG cluster during session 2 compared to session 3 (mean difference = 0.603, *t*(12) = 2.791, *p* < *0.05*). None of the interaction effects reached significance for the left lateral inferior pre-CG (all *F* ≤ 4.030).

## Discussion

In this longitudinal fMRI study, we investigated developmental changes in cortical responses to audio-visual letter-speech sound stimuli using a text-based recalibration paradigm in children aged 8–9 to 10–11 years across three measurement time points spaced ~1 year apart. Our fMRI results showed a significant developmental change in cortical activation in left hemisphere STG and lateral inferior pre-CG regions. The changes in cortical activation across sessions within the left STG cluster were associated with children’s age during session 1 as well as the strength of the behavioural recalibration effect. No links with children’s reading fluency or phonological processing (pre-test slope steepness) were observed.

All children successfully performed the text-based recalibration task, as reflected by the different /aba/ response proportions to the ambiguous post-test sounds following each type of exposure block. However, as in our previous study in 8–10 year-old children, these behavioural recalibration effects did not reach significance in the MRI scanner environment^46^, while children in this age range did show significant text-based recalibration in an offline behavioural study^44^. This discrepancy can likely be attributed to the acoustic noise, and/or altered (visual) attentional focus in MRI environment^47,48^. By comparison, typically reading adults have been reported to show reliable recalibration effects both in and out of the MRI scanner^42,49,50^. Similarly to our previous fMRI study^46^, a sub-group of children in the current sample did show recalibration in the MRI scanner (9 in session 1, 8 in session 2 and 11 in session 3). It is therefore likely that the recalibration effect may emerge in the MRI environment as children get older, although this would need to be confirmed in future studies.

To investigate cortical activation associated with audio-visual processing of letters and speech sounds and its potential links to behavioural and reading measures, we focused our fMRI analyses on the audio-visual exposure blocks of the recalibration task. Our results showed bilateral cortical activation patterns across the sessions in brain areas previously linked to audio-visual integration and reading^7,28,29,31,32,51^, as well as to text-based recalibration specifically^42,46^. These brain areas included bilateral STG, occipital and parietal areas as well as the left frontal cortex. Comparisons of brain activation between sessions revealed longitudinal changes in activation in left hemisphere STG and lateral inferior pre-CG clusters. Plotting children’s average individual brain activation across sessions within these clusters revealed substantial variability with mostly non-linear activation changes. Post-hoc mixed regression analysis showed that activation in the left STG cluster was associated with children’s baseline age. Namely, children who were younger during session 1 showed higher left STG activation during session 2 compared to session 3, whereas older children showed more activation in session 3 compared to session 2. Visual inspection of changes in activation across sessions for the younger vs. the older children, suggested that younger children, aged 8 years during the first measurement session, were more likely to show a non-linear, inverted-u-type activity change across sessions. Older children, aged 9 years during the first session, instead showed a more individually variable, but on average smaller linear change in activation. These results will need to be verified in a larger sample of children.

Non-linear (e.g. inverted-u) developmental changes are reminiscent of cross-sectional results reported in adults and children of different age groups in previous electroencephalography (EEG) studies employing an audio-visual oddball paradigm with letters and speech sounds. These studies have indicated audio-visual deviancy responses in two time windows—a mismatch negativity response (MMN) around 100–250 ms, associated with automatic processing of audio-visual letter-speech sound stimuli^5,52,53^ and a sustained late negativity (LN) response associated with more “effortful” and deliberate integration mechanisms^4,5,19^. Using this paradigm, 8–9 year-old typically reading children were observed to have a relatively wide audio-visual integration window and more pronounced audio-visual MMN and LN responses^5^ compared to both younger and older children^4^ as well as adults^52^. That is, the findings reveal an apparent evolution from an LN-only response in 7/8-year olds to both MMN and LN responses and a broad audio-visual integration window in the 8/9-year olds, followed by increasing selectivity across 11-year olds and adults. This pattern of results arguably reflects a switch from more effortful to automatised audio-visual processing, with fully automatised letter-speech sound processing in adults. The observed longitudinal changes in left STG activation to letter-speech sound pairs across sessions may represent a different neural correlate of the same developmental trajectory from effortful letter-for-letter mapping of text and speech towards quick and automatic processing of over-learnt letter-speech sound associations.

Studies investigating text specialisation and sensitivity using both EEG and fMRI have shown similar non-linear (inverted-u) activation patterns of developmental change within ventral occipito-temporal cortex and the VWFA in particular^14,16,18,54,55^, paralleled by a change in grey matter volume of the left occipito-temporal sulcus^12^. The fact that we did not observe significant session differences in ventral occipito-temporal cortex activation in the current sample may be due to task characteristics or age group differences. The recalibration task employs only two types of simple visual text stimuli that do not require extensive processing or explicit judgment (“aba” and “ada”). It is therefore likely that this paradigm does not provide optimal engagement of the VWFA, making it less suitable for exploration of VWFA activation changes over time. The exposure blocks of text-based recalibration combine these clear visual text stimuli with an ambiguous speech sound. The combination of the two modalities likely produces different auditory percepts across trials. This could result in an enhancement of activation in speech sensitive STG regions, that have been shown to encode these type of perceptual changes^42,49,56,57^, especially at the age where letter-sound associations are in the process of becoming automatised. In line with this interpretation, our mixed regression analysis indicated that, regardless of age, children who did show (stronger) recalibration effects in the MRI scanner across sessions, showed more left STG activation during session 2 compared to session 3. It is likely that this finding is linked to an emergence and/or strengthening of the effect across sessions, as 9 children showed recalibration in session 1, 8 children in session 2 and 11 children in session 3. Our previous fMRI study using the text-based recalibration paradigm in children with and without dyslexia showed a correlation between letter-speech sound identification scores and left STG activation across children, with lower activation in the region linked to better task performance^46^. The observed decrease in left STG activation between session 2 and 3 in children who show a recalibration effect may similarly represent successful mapping of the text and ambiguous speech stimuli leading to (stronger) behavioural recalibration.

Beyond changes specific to audio-visual integration of letters and speech sounds, reading and spoken language development may mutually lead to the refinement of cortical responses to speech during reading development. It has been proposed that learning to read in an alphabetic script in particular, alters phonological processing by magnifying the phonological representations of the smallest-possible visual unit (often a single letter) and is accompanied by a developmental increase in STG responses observed during auditory rhyme judgement tasks in alphabetic compared to logographic orthographies^58^. These reading-induced changes in speech perception may further affect categorical perception of phonemes^59^ and interact with reading skills^60^. Developmentally, categorical perception of phonemes has been associated with left-lateralized activation of posterior STG that scales with increasingly distinct definition of phoneme categories in 7–12 year-old children^60^. Furthermore, the STG shows distinct phoneme-category specific activation patterns following categorical learning and subsequent perception of novel speech sounds^61^, as well as task-dependent^56,57^ and recalibration-induced^42,49^ changes in speech sound representation.

The emergence of the left lateral inferior pre-CG as one of the regions showing significant session effects is in line with this interpretation. Previous research has shown cortical activation in frontal/motor speech production areas during speech perception tasks (e.g.^62–65^) and audio-visual speech disambiguation^66^, particularly when using noisy/degraded auditory stimuli^67–70^. The lateral inferior pre-CG cluster in the current study overlaps with the somatosensory lip and tongue representation^71^. An fMRI study investigating the presence of articulatory information during auditory-only speech perception has shown that activity patterns within this brain area, albeit in the right hemisphere, encode information on the place of articulation of speech sounds^72^. The developmental change in cortical activation in this region may thus result from the fact that children differentially engaged the lateral pre-CG to help disambiguate the ambiguous speech stimuli, which in fact were taken from an /aba/-/ada/ continuum involving a change in place of articulation. On a more general level, this activity could relate to changes in subvocal rehearsal of the perceived /aba/ vs. /ada/ sounds.

As children grow and learn, the exact timing of developmental turning points related to e.g. learning phases in acquiring solid letter-speech sound mappings, refinement of speech perception and visual text processing, likely varies per child. It has been shown that children reach developmental milestones at different times^73,74^. Changes in cortical activation associated with these transitions may vary per brain region or involve different brain networks depending on the individual and behavioural goal. This may explain the observed individual differences in cortical activation changes across sessions within the left STG and lateral inferior pre-CG (grey lines, Fig. 4). To further understand these individual differences, it will be important to extend and replicate the current developmental findings in future longitudinal studies including larger sample sizes and, for example, multiple reading/language tasks. These studies may reveal task- and region-dependent developmental trajectories that, ultimately, will explain (a)typical variability in children’s reading development.

In terms of underlying developmental mechanisms of change, non-linear, inverted-u-type trajectories are reminiscent of those associated with children’s skill learning. When learning a skill, children benefit from both active strategies (e.g. explicitly mapping letters and speech sounds) as well as passive mechanisms such as statistical and associative learning^75–77^. The use of active strategies during the early stages of learning serves as input for the passive mechanisms and the dynamics of the two change over the course of skill acquisition. Namely, with increased proficiency, children begin to rely less on the explicit mechanisms and instead switch to fast, automatised processing supported by passive mechanisms such as associative learning^78^. In fact, skill learning in general is thought to reflect an expansion-renormalisation pattern, with an initial increase in cortical activation/volume in the key areas involved in the acquisition of a specific skill, followed by a decrease once proficiency is reached^79,80^. Because reading is a culturally acquired skill, similar mechanisms may underlie the non-linear developmental patterns in our left STG and lateral inferior pre-CG results. In particular, they may reflect changes during the proposed prolonged time-course of letter-speech sound automatisation^1,4^, which in case of the lateral inferior pre-CG may specifically be related to the differential engagement of the region to disambiguate the audio-visual text-ambiguous speech stimuli used in our task.

In conclusion, the observed longitudinal changes in 8-11 year-old children’s left STG and lateral inferior pre-CG responses to speech sounds and letters, together with previous findings in higher order visual areas, point to regionally specific functional changes in the developing reading and speech perception networks. Our findings show non-linear developmental trajectories in a speech (and text) sensitive auditory cortical region, and may reflect the fine-tuning of letter-speech sound mappings representing a gradual switch from more effortful to automatised processing. The observed changes in lateral inferior pre-CG activation may in turn point to a specific role of motor articulatory functions in audio-visual speech disambiguation during text-based recalibration. Future longitudinal studies with larger sample sizes are needed to verify the observed pattern of responses within these areas and the reading network as a whole and to establish their link to individual differences in typical as well as atypical reading development. These studies could also use tailored fMRI designs to investigate developmental changes in text-induced shifts in auditory cortical representations of the ambiguous post-tests sounds using a multivariate decoding approach (see^42^). Relating individual decoding accuracies of /aba/ vs. /ada/ percepts to children’s reading skills could provide further insights into the links between reading gains and reading-induced changes in the auditory cortical representation of speech sounds.

## Methods

### Participants

A total of 43 children (mean age at time-point 1: 8.9 ± 0.7 years; 24 females) were recruited from local elementary schools in the Maastricht area in the Netherlands. We included children of various reading levels ranging from poor to excellent. None of the children had persistent reading problems that would meet the criteria for a dyslexia diagnosis. Of the 43 children, 7 completed only the first measurement, 16 completed only the first 2 measurements, and the remaining 20 participants participated in all 3 measurements of the given study. Note that the relatively large number of children who dropped out after the 2nd measurement was due to an almost 4 month closure of the MRI research facilities during the first Covid-19 lock-down (March–June 2020). Of the 20 participants, one was excluded from the analyses due to poor data quality in session 3, and one participant had undergone remediation for dyslexia. The final sample consisted of 18 children who participated in all three longitudinal measurements (mean age at time-point 1: 8.7 ± 0.6 years; 11 females; 1 left-handed). All fMRI and post-hoc analyses were conducted on this sample of 18 children. The average time between the first two measurements was 13 months (±1.7 months; range 11–17 months; mean age at time-point 2: 9.8 ± 0.6 years) and the average time between measurements 2 and 3 was 10.4 months (±1.9 months; range 6–15 months; mean age time-point 3: 10.6 ± 0.59 years). The shorter time period between measurements 2 and 3 was driven by 5 participants who were scanned on average 8 months after the second measurement (range 6–9 months) due to planned orthodontic treatment that would have resulted in discontinuation of research participation. All children were native Dutch speakers, had no reported hearing impairments, normal or corrected-to normal vision and no history of developmental or neurological disorders. All children received a present (in session 1) or a gift card (sessions 2 and 3) for participation, along with a small, customised gift per scanning session. Parents provided written informed consent prior to each measurement in accordance with the declaration of Helsinki. The study was approved by the ethics committee of the Faculty of Psychology and Neuroscience, Maastricht University.

### Literacy and cognitive skills

After each scanning session, the children completed computerised tasks of the 3DM test battery (Dyslexia Differential Diagnosis^81^; assessing reading and phonological skills. In addition, at first and last measurements, the children completed two sub-tests of the Dutch version of the Wechsler Intelligence Scale for Children-III (WISC-III-NL^82^; –verbal (similarities) and non-verbal (block design). The subsequent sub-tests of the 3DM were used in the present study: reading, letter-speech sound identification and phoneme deletion (elision).

The reading task was divided into three sections: reading of high-frequency, low-frequency and pseudo words. The total duration of the task was 90 s (30 s per category) and the children were instructed to read as quickly and accurately as possible. Reading fluency was calculated as the total number of words read within the time limit. During the letter-speech sound identification task, the children were presented with a speech sound aurally via headphones and asked to indicate the corresponding letter(s) out of four possibilities presented on the computer screen, by button press. The letter-speech sound identification accuracy score was calculated as the percent of correctly identified items out of the total number of completed items (maximum items included in the task was 90). The phoneme deletion task consisted of 23 aurally presented pseudo-words, followed by an aurally presented phoneme. The children were instructed to repeat the pseudo-word without the phoneme (e.g. say /tesk/ without the /s/ sound). The accuracy score for this task was calculated as the percent of correctly identified items out of the total number of completed items, with an upper response time limit of 15 s per item. All task instructions were simultaneously presented aurally over headphones and visually on the computer screen. The children were instructed to perform all tasks as quickly and accurately as possible. Descriptive statistics of the sample and the reading scores (raw scores and age-normed t-scores) across sessions are reported in Table 2. One of the children showed a low t-score for reading fluency (*t* = 30) and another child for the phoneme deletion accuracy (*t* = 26) in session 1. While the first child’s reading fluency t-scores remained stable across the three measurement sessions, their phoneme deletion scores were variable and ranged from low average to slightly below average. The child with a low phoneme deletion task score in session 1 showed similar t-scores for this task across the 3 sessions, ranging from very poor to poor, while their raw scores improved. Similarly, this child’s reading scores improved from below average to (low) average. Boxplots analyses of the raw- and t-scores of these tasks did not indicate any outliers. Thus, the children’s reading and phoneme deletion scores did not significantly differ from the overall range of scores in the current sample.

**Table 2:** Descriptive statistics of the sample.

	Session 1		Session 2		Session 3		Between session comparison	
	Mean	Range	Mean	Range	Mean	Range	*F*(2,30)	*p*
Age (years)	8.75	8–9.7	9.88	9.1–11	10.68	9.9–11.6	–	–
Reading fluency (raw)^b^	113.72	74–178	129.29	96–181	137.53	99–201	20.68^a^	0.001
Reading fluency (t)^c^	52.27	30–80	53.17	32–80	53.12	30–80	0.12	0.890
Letter-speech sound identification fluency (raw)	42.72	39–45	42.29	38–45	42.58	39–45	0.36	0.702
Letter-speech sound identification fluency (t)	54.78	48–73	58.06	39–68	56.70	40–72	0.79	0.460
Phoneme deletion fluency (raw)	17.05	2–23	17.59	9–23	19.41	10–23	3.07	0.061
Phoneme deletion fluency (t)	51.72	0–70	58.59	37–80	57.29	29–71	2.89	0.071
WISC Verbal (Similarities)^d^	15.22	12–18	–	–	15.06	10–19	–	–
WISC Non-verbal (block design)^d^	12.78	9–19	–	–	11.88	6–17	–	–

^a^Greenhouse-Geisser corrected F(1,21).

^b^Raw scores, number of correct items across three sub-groups (high-frequency, low-frequency and pseudo words) per 90 s, number of correct responses out of 90 items (letter-speech sound identification) or 28 items (phoneme deletion).

^c^t-Scores, age-appropriate norm scores mean 50, SD = 10.

^d^Age-appropriate norm scores mean 10, SD = 3.

### Stimuli

The auditory stimuli employed in the recalibration task consisted of 650 ms recordings of the speech sounds /aba/ and /ada/ spoken by a male native Dutch speaker (see^37^ for a detailed description). The speech sounds were used to create a nine-token sound continuum ranging from a clear /aba/ sound to a clear /ada/ sound, with 7 ambiguous sounds in between, by changing the second formant (F2) in eight steps of 39 Mel using PRAAT software^83^. The visual stimuli consisted of corresponding “aba” and “ada” text presented in white at the centre of a black screen in ‘Times New Roman’ font (font size 50). The auditory and visual stimuli were presented using Presentation software (Version 18.1, Neurobehavioral Systems, Inc., Berkeley, CA, United States).

### Experimental design and procedure

All testing sessions started with a practice round in a mock scanner, followed by the actual (f)MRI scans, and ended with literacy and cognitive skill testing. During the mock scanner practice, the children were introduced to the scanner environment, including the use of in-scanner MR compatible headphones (Sensimetrics, model S14, www.sens.com) and button boxes, as well as the types of scanner sounds they would hear. At the beginning of the practice session, the children were reminded of how to perform the Recalibration task, and the scanning procedure and duration were explained. The children then completed a pre-test (see below), practiced one round of the Recalibration task and completed motion training, in order to improve later (f)MRI data quality. During motion training, an elastic headband containing a motion tracker was placed on the child’s forehead. The sensor was calibrated to indicate when motion along the horizontal and/or vertical plane exceeded 2 degrees and linked to an in-house movie player software that was used to play a cartoon. As soon as head motion exceeded the 2 degree threshold, the cartoon paused and shrank until the child was lying still again. This helped illustrate how still the children should aim to lie in the real MRI scanner while doing the tasks and to get them used to the MR environment. The mock scanner session lasted ~15–20 min. The children subsequently took part in a 1 h 15 min MRI session. While the total time required for the acquisition of the functional and structural images was 45 min, we took the time to make sure the children were comfortable when preparing to go into the scanner and allowed for short breaks between each anatomical/structural scan to avoid fatigue. After the MRI session, the children completed the reading and phonological tasks in sessions 1, 2 and 3 (±20 min) and the two subtests of WISC-NL-III in sessions 1 and 3 (±20 min). The total testing time was ~2 h 15 min. In addition, children had 2 breaks—one after completing the mock scanner practice round, and one after the MRI scan before completing the reading and cognitive tasks.

### Pre-test

**Fig. 5:**
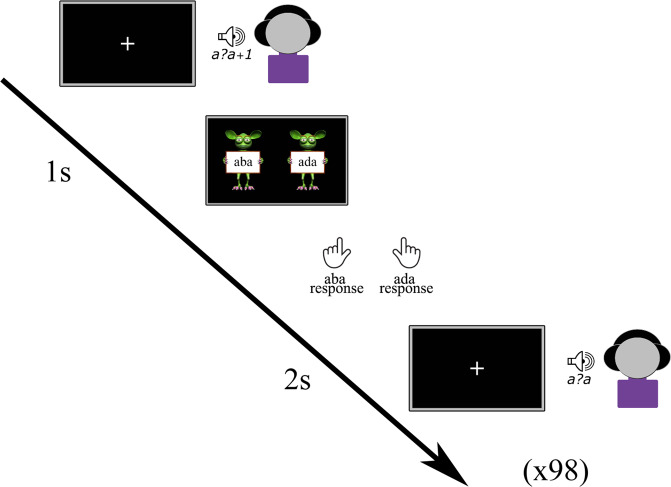
Pre-test. Each auditory trial was followed by a response cue. Once a response was provided, the subsequent trial was presented.

During the mock scanner session, children completed a pre-test, in which they heard each of the 9 sound tokens along the /aba/-/ada/ continuum a total of 98 times in a randomised order, with the middle sounds along the continuum presented more frequently than the two clear tokens^37,49,84^. The children were instructed to listen carefully to each sound and to indicate if they perceived it as /aba/ or as /ada/ by pressing the left or right innermost button of the MR compatible button box with their left/right index finger following a response cue. The response cues consisted of text “aba” (left) and “ada” (right), held up by cartoon monsters created using the Monster Workshop content pack of the iClone 6 software (https://www.reallusion.com/). During the presentation of the speech sounds, the children viewed a black screen with a white fixation cross followed by the response cue 1 s later. Each trial was terminated after the child provided a response, triggering the presentation of the subsequent speech sound after 2 s (Fig. 5). The total duration of the pre-test was ~5 min.

The results of the pre-test were used to individually determine the most ambiguous speech sound along the /aba/–/ada/ continuum for each participant, based on the proportion of /aba/ responses to each sound token. The most ambiguous sound was identified as the token with an /aba/ vs. /ada/ response proportion closest to 0.5, representing the phoneme boundary^44,84^. This individually determined most ambiguous sound was used in the audio-visual exposure blocks and post-test trials of the recalibration task. In addition to the most ambiguous /a?a/ sound, the post-test trials also included its two flanking sounds along the /aba/-/ada/ continuum, namely /a?a/+1 and /a?a/-1. In addition, the resulting sigmoidal curve showing the response proportions to each sound token, served as an indicator of children’s categorical perception of the phonemes /aba/ and /ada/, with a steeper slope representing a sharper distinction between phoneme categories.

### Recalibration task

**Fig. 6:**
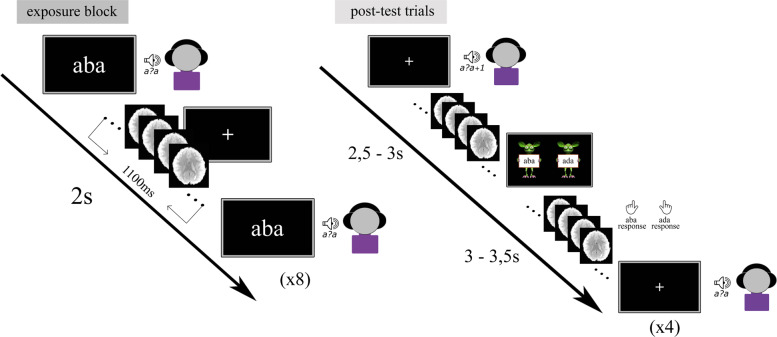
Text-based recalibration paradigm in the MRI environment. Set up of the audio-visual exposure blocks (left) and subsequent auditory-only post-test trials (right).

The recalibration task consisted of audio-visual exposure blocks followed by auditory post-test trials (Fig. 6). During each exposure block, the children were presented with either the text “aba” or “ada” in combination with the individually determined most ambiguous speech sound /a?a/ 8 times. The audio-visual stimuli were presented simultaneously (relative SOA of 0 ms), the duration of the auditory stimuli was 650 ms and visual text was presented for 1 s. The inter-trial interval between subsequent audio-visual exposure trials was set to 2 s (1 TR). The /aba/ and /ada/ exposure blocks were presented in a pseudo-randomised order, making sure that the same type of exposure block was not repeated more than twice in a row. During the audio-visual exposure blocks, the children were instructed to pay close attention to the speech sounds and text without providing a response.

Each exposure block was followed by four auditory-only post-test trials, the onset of which was jittered to 10 s (4–6 TR). These jittered periods served as the baseline condition, during which the children fixated on a white cross in the middle of a black screen. The post-test trials were presented in a randomised order with the most ambiguous sound /a?a/ presented twice, and each of the flanking sounds /a?a/+1 and /a?a/-1 on the /aba/-/ada/ continuum presented once. Following each post-test sound, a response cue containing cartoon monsters appeared on the screen. The duration of the response cue was fixed to 3 s and the onset was jittered 2,5–3 s with respect to the post-test sound. The subsequent post-test trial was presented 3–3,5 s following the response cue. The total inter trial interval (ITI) between post-test trials was 6 s (3 TR). Children were instructed to listen carefully to each post-test sound and to respond whether they perceived it as /aba/ or as /ada/ upon the presentation of the response cue using the MR-compatible button boxes. The responses were made by pressing the innermost button of the button box with the left/right index finger, as practiced in the mock scanner.

Children completed a total of four runs of the recalibration task, corresponding to 24 audio-visual exposure blocks (12 with “aba” text and 12 with “ada” text) and 4*24 post-test trials. The recalibration effect was calculated as the difference in average /aba/ response proportions across the post-test sounds following the two types of exposure blocks (i.e. total /aba/ response proportion after an “aba” exposure block minus total /aba/ response proportion following an “ada” exposure block). All auditory and audio-visual stimuli were presented during a 900 ms silent gap between volume acquisitions.

### Statistical analyses behavioural data

Children’s performance on the pre-test and recalibration tasks was analysed using repeated measures (RM) ANCOVA. The RM ANCOVA analyses for the pre-test included within-subject factors Session (1, 2, 3) and sound (1–9) and baseline age as a covariate. The RM ANCOVA for the Recalibration task was performed on the /aba/ response proportions following each of the three post-test sounds and consisted of within-subject factors: session (1, 2, 3), exposure type (“aba” vs. “ada”) and post-test sounds (/a?a/-1, /a?a/, /a?a/+1) with baseline age as a covariate. The degrees of freedom were adjusted using the Greenhouse-Geisser correction for conditions, which violated the sphericity assumption. Prior to running the analyses, the data were assessed for outliers using boxplots. This amounted to four observations for the pre-test (two outliers—lower quartile plus 1.5 times inter-quartile range and two extreme outliers—lower quartile plus 3 times inter-quartile range) and one observation for the recalibration analyses (upper quartile plus 1.5 times inter-quartile range). For both analyses, the observations identified as outliers and extreme outliers were excluded.

We used a custom logistic function described in^44^ (Eq. (1)) and the Slope Fitting tool in MATLAB 2019a (The MathWorks, Inc., Natick, MA, United States) to assess the fit of each subject-specific pre-test sigmoidal curve resulting from children’s /aba/ response proportions to each of the 9 sounds along the /aba/-/ada/ continuum (see Fig. 1). This was done to obtain the slope value (c in eq. (1)) of the sigmoidal response curve as it represents the distinction between the /aba/ and /ada/ phoneme categories. Namely, the closer this value was to 0, the steeper the slope and sharper the phoneme category distinction. The pre-test slope value providing the best fit to the data was obtained using the MATLAB least squares solver. To optimise the outcome, the variables in eq. (1) were restricted to the following values: 0 ≤ a ≥ 10, −10 ≤ b ≥ 10, −10 ≤ c ≥ 10, −9 ≤ d ≥ 18 and the best fit was determined after 30 iterations of the procedure.

$${{{\mathrm{y}}}} = \frac{{{{\mathrm{a}}}}}{{1 + e^{\frac{{ - \left( {{{{\mathrm{x}}}} - {{{\mathrm{d}}}}} \right)}}{{{{\mathrm{c}}}}}}}} + {{{\mathrm{b}}}}{{{\mathrm{.}}}}$$

Equation 1: a = amplitude; b = lowest asymptote of y-axis; c = slope of the function; d = location of the category boundary.

### (f)MRI measurements

Brain Imaging was performed with a Siemens Prisma 3 T MRI scanner (Siemens Medical Systems, Erlangen, Germany) using a 64-channel head–neck coil. Five functional runs were acquired (2.5 mm × 2.5 mm × 2.5 mm resolution) with a multi-band factor of five echoplanar-imaging sequence (repetition time [TR] = 2000 ms, acquisition time [TA] = 1100 ms, field of view = 210 mm × 210 mm, echo time [TE] = 35.8 ms). Each volume consisted of 50 slices (no gap), covering the whole brain. The recalibration task consisted of four 5 min runs, followed by a 7 min localiser task not included in the current analyses. The speech stimuli were presented binaurally at a comfortable listening level via MR compatible headphones (Sensimetrics, model S14, www.sens.com), in the 900 ms silent gap between two consecutive volume acquisitions. In addition, a high-resolution structural scan (1 mm × 1 mm × 1 mm) using a T1-weighted three-dimensional MPRAGE sequence ([TR] = 2300 ms, [TE] = 2.98 ms, 192 sagittal slices) was acquired.

### (f)MRI pre-processing

Data pre-processing and analyses were performed using BrainVoyager QX version 2.8 and BrainVoyager versions 20.6 and 21.4 (Brain Innovation, Maastricht, The Netherlands) as well as custom MATLAB routines (The MathWorks, Inc., Natick, MA, United States). The functional data were aligned to the first volume of the first functional run and underwent 3D motion correction (trilinear sinc interpolation), slice scan time correction and high pass temporal filtering (five cycles per time course). The anatomical data underwent manual inhomogeneity correction to improve white matter-grey matter boundary segmentation and was transformed into Talairach space^85^. To ensure all anatomical runs were well aligned across sessions, the native space anatomical data of sessions 2 and 3 was coregistered to the ACPC transformed session 1 anatomy using vmr-vmr co-registration in Brainvoyager QX. The resulting transformation file was subsequently applied to the anatomical runs of sessions 2 and 3 resulting in well aligned anatomical data. Transformation to Talairach space was performed using the transformation file of session 1.

The functional data were subsequently aligned across sessions by co-registration to the Talairach transformed anatomical data of session 1, re-sampled to 3 mm iso-voxel resolution and spatially smoothed using a 6 mm FWHM Gaussian kernel. Volumes of functional runs affected by excessive head motion (≥3 mm/degree translation/rotation in any direction) were removed from the run. If the number of affected volumes exceeded 20%, the run was excluded from further analyses. This amounted to a total of five runs in the second session and six runs in the third session. In addition, not all children completed all four recalibration runs due to time constraints. The total number of runs not acquired across all three sessions was 8 (2 in session 1 and 3 in sessions 2 and 3). The final number of runs included in the analyses was 197: 70 session 1, 64 session 2, 63 session 3.

Individual cortical surface representations of session 1 anatomical scans were automatically constructed for each participant based on the white matter-grey matter boundary. The boundary was then manually adjusted, and aligned using cortex based alignment employing a moving-target group average based on curvature information, resulting in an anatomically-aligned group-average 3D cortical representation^86^. Each participant’s functional data were projected onto their cortical surface to create surface-based time courses. All functional data were subsequently analysed per hemisphere at the surface level using the group-aligned average cortical surfaces.

### Whole brain univariate fMRI analysis

Cortical activation across sessions was assessed using a random effects (RFX) general linear model (GLM) approach based on the individual surface-based time courses. The GLM included a predictor for each type of exposure block (“aba” and “ada”), predictors for each post-test sound (4 predictors) and z-transformed motion predictors as variables of no interest to improve the signal-to-noise ratio in the data. Cortical activation in response to the audio-visual letter-ambiguous speech stimuli for each measurement session was assessed using contrast maps (t-statistics) based on the results of the GLM model. The contrast maps compared cortical activation during the exposure blocks (“aba” + “ada” exposure blocks; EXP) and the fixation cross baseline and were corrected for multiple comparisons using a whole-brain FDR threshold of *q* < 0.05. To explore the longitudinal changes in cortical activation across sessions, in a next step we performed a RM ANOVA with session as the within-subject factor. This yielded a cortical map (F-statistic) of areas that show significant changes in activation across the three measurement sessions at a whole-brain FDR threshold of *q* < 0.05. To limit our results to brain regions that showed significant activation during the audio-visual exposure blocks, the resulting F-map was masked with the combined activation map (EXP > baseline; FDR *q* < 0.05) across the three sessions. Thus, only the regions that showed overlap with the mask were explored further with post-hoc paired-samples *t*-tests for pairwise activation differences (individual average beta values EXP > baseline) between sessions 1 & 2, 2 & 3 and 1 & 3. An FDR correction for multiple comparisons (using the Benjamini and Hochberg procedure) was performed on the *p* values of the post-hoc paired-samples *t*-tests using MATLAB.

### Multiple regression analysis

Post-hoc mixed regression analyses were performed to explore possible relations between behavioural measures and the observed changes (main effect of Session) in cortical activation in the left STG and lateral inferior precentral gyrus (SPSS v26, IBM Corp., Armonk, NY, United States). We opted for this approach due to its reported suitability for accelerated longitudinal designs and tolerance for handling missing data^87,88^. We constructed two marginal models including each child’s average beta values for each cluster as the dependent variable, within-subject factor session, and within-subject covariates: baseline age, reading fluency, recalibration effect and pre-test slope value, as well as interactions between session and each of the covariates. To avoid circularity, we do not interpret the main effect of session, as this is already established by the RM ANOVA results, and only interpret the interactions between session and the covariates. The letter-speech sound identification and phoneme deletion fluency scores were not included in the analysis as they did not show much variation across sessions (see Table 2). Prior to running the model, the data were assessed for outliers using boxplots. Six observations in total were identified as outliers/extreme outliers and excluded from the analyses. Four observations were outliers—two upper quartile plus 1.5 times inter-quartile range (one value for reading fluency, one for the recalibration effect) and two lower quartile plus 1.5 times inter-quartile range (both pre-test slope values). Two additional pre-test slope values were categorised as extreme outliers, i.e. lower quartile plus 3 times inter-quartile range. In addition, four observations had missing data—two pre-test slope values and two reading fluency measures. The final models reported were Fixed-effects models estimated using Restricted Maximum Likelihood and an unstructured covariance matrix for RM. Model residuals had a normal distribution and showed no heteroscedasticity.

### Reporting summary

Further information on research design is available in the Nature Research Reporting Summary linked to this article.

## Supplementary information

Reporting Summary

## Data Availability

Anonymised raw fMRI and behavioural data of the children for whom parents have given (anonymised) data sharing consent are available from the corresponding author upon reasonable request.

## Code availability

All code used for task presentation and data analysis is available from the corresponding author.
