# Supplementary material for: Longitudinal changes in cortical responses to letter-speech sound stimuli in 8–11 year-old children
Source: NPJ Sci Learn. 2022 Jan 25;7:2. doi: 10.1038/s41539-021-00118-3 (PMC8789908; doi:10.1038/s41539-021-00118-3)
Supplement: Supplementary file 1 — Reporting Summary [file 41539_2021_118_MOESM1_ESM.pdf]

nature portfolio

Corresponding author(s): Linda Romanovska

Last updated by author(s): Dec 13, 2021

Reporting Summary

Nature Portfolio wishes to improve the reproducibility of the work that we publish. This form provides structure for consistency and transparency in reporting. For further information on Nature Portfolio policies, see our Editorial Policies and the Editorial Policy Checklist.

Statistics

For all statistical analyses, confirm that the following items are present in the figure legend, table legend, main text, or Methods section.

n/a	Confirmed	
☐	☒	The exact sample size (n) for each experimental group/condition, given as a discrete number and unit of measurement
☐	☒	A statement on whether measurements were taken from distinct samples or whether the same sample was measured repeatedly
☐	☒	The statistical test(s) used AND whether they are one- or two-sided<br>*Only common tests should be described solely by name; describe more complex techniques in the Methods section.*
☐	☒	A description of all covariates tested
☐	☒	A description of any assumptions or corrections, such as tests of normality and adjustment for multiple comparisons
☐	☒	A full description of the statistical parameters including central tendency (e.g. means) or other basic estimates (e.g. regression coefficient) AND variation (e.g. standard deviation) or associated estimates of uncertainty (e.g. confidence intervals)
☐	☒	For null hypothesis testing, the test statistic (e.g. F, t, r) with confidence intervals, effect sizes, degrees of freedom and P value noted<br>*Give P values as exact values whenever suitable.*
☒	☐	For Bayesian analysis, information on the choice of priors and Markov chain Monte Carlo settings
☒	☐	For hierarchical and complex designs, identification of the appropriate level for tests and full reporting of outcomes
☒	☐	Estimates of effect sizes (e.g. Cohen's d, Pearson's r), indicating how they were calculated

Our web collection on statistics for biologists contains articles on many of the points above.

Software and code

Policy information about availability of computer code

Data collection	Presentation Software version 18.1 Neurobehavioral Systems, Inc., Berkeley, CA, United States; 3DM Differential Diagnostics for Dyslexia: Cognitive Analysis of Reading and Spelling. Amsterdam: Boom Test Publishers; 2009
Data analysis	BrainVoyager QX version 2.8 and BrainVoyager versions 20.6 and 21.4 (Brain Innovation, Maastricht, The Netherlands); MATLAB version R2019a (The MathWorks, Inc., Natick, MA, United States)

For manuscripts utilizing custom algorithms or software that are central to the research but not yet described in published literature, software must be made available to editors and reviewers. We strongly encourage code deposition in a community repository (e.g. GitHub). See the Nature Portfolio guidelines for submitting code & software for further information.

Data

Policy information about availability of data

All manuscripts must include a data availability statement. This statement should provide the following information, where applicable:

- Accession codes, unique identifiers, or web links for publicly available datasets
- A description of any restrictions on data availability
- For clinical datasets or third party data, please ensure that the statement adheres to our policy

A subset of data sets for which parents have given data sharing consent are available from the corresponding author upon reasonable request.

Field-specific reporting

Please select the one below that is the best fit for your research. If you are not sure, read the appropriate sections before making your selection.

☒ Life sciences ☐ Behavioural & social sciences ☐ Ecological, evolutionary & environmental sciences

For a reference copy of the document with all sections, see nature.com/documents/nr-reporting-summary-flat.pdf

Life sciences study design

All studies must disclose on these points even when the disclosure is negative.

Sample size	Participants who completed all 3 annual fMRI measurement sessions.
Data exclusions	Missing or poor quality data in sessions 2 or 3 and past dyslexia diagnosis (1 participant)
Replication	The fMRI and behavioural findings of the current manuscript replicate our previous fMRI study in children with and without dyslexia. Future longitudinal studies with larger sample sizes are needed to test the reproducibility of the current findings.
Randomization	n/a
Blinding	n/a

Reporting for specific materials, systems and methods

We require information from authors about some types of materials, experimental systems and methods used in many studies. Here, indicate whether each material, system or method listed is relevant to your study. If you are not sure if a list item applies to your research, read the appropriate section before selecting a response.

Materials & experimental systems

n/a	Involved in the study	
☒	☐	Antibodies
☒	☐	Eukaryotic cell lines
☒	☐	Palaeontology and archaeology
☒	☐	Animals and other organisms
☐	☒	Human research participants
☒	☐	Clinical data
☒	☐	Dual use research of concern

Methods

n/a	Involved in the study	
☒	☐	ChIP-seq
☒	☐	Flow cytometry
☐	☒	MRI-based neuroimaging

Human research participants

Policy information about studies involving human research participants

Population characteristics	Children of varying reading abilities who have not received a dyslexia diagnosis aged 8 - 11 years, 11 females.
Recruitment	Participants were recruited from local primary schools in the area.
Ethics oversight	Maastricht University

Note that full information on the approval of the study protocol must also be provided in the manuscript.

Magnetic resonance imaging

Experimental design

Design type	Block design
Design specifications	Each fMRI measurement session consisted of 5 runs and an anatomical scan. The recalibration task (reported in this study) consisted of four 5 minute runs, followed by a 7-minute localizer task not included in the current analyses. Each of the 4 recalibration task runs included 6 exposure blocks (3 "aba" and 3 "ada"). During each exposure block, the children were presented with either the text “aba” or “ada” in combination with individually determined ambiguous speech sound /a?a/ 8 times. The audio-visual stimuli were presented simultaneously (relative SOA of 0 ms), the duration of the auditory stimuli was 650 ms and visual text was presented for 1s. The inter-trial interval between subsequent audio-visual exposure trials was set to 2 s (1 TR). The /aba/ and /ada/ exposure blocks were presented in a pseudo-

randomised order, making sure that the same type of exposure block was not repeated more than twice in a row. Each exposure block was followed by four auditory-only post-test trials, the onset of which was jittered to 10s (4-6 TR). These jittered periods served as the baseline condition, during which the children fixated on a white cross in the middle of a black screen. Following each post-test sound, a response cue containing cartoon monsters appeared on the screen. The duration of the response cue was fixed to 3 s and the onset was jittered 2,5 - 3 s with respect to the post-test sound. The subsequent post-test trial was presented 3 - 3,5 s following the response cue. The total inter trial interval (ITI) between post-test trials was 6 s (3 TR).

Behavioral performance measures

We record children's /aba/ and /ada/ responses following each auditory-only post-test sound and calculate the overall / aba/ response proportion for each of the sounds to represent the behavioural task performance. Statistical analyses assessing the significance of the behavioural recalibraiton effect were conducted using repeated measures ANCOVA including baseline age in months as a covariate. Reading skill and WISC sub-test scores are described in the manuscript. Changes in these scores across measures were assessed with RM ANOVA.

Acquisition

Imaging type(s)

Functional and structural

Field strength

3T

Sequence & imaging parameters

The functional runs were acquired (2,5 mm x 2,5 mm x 2,5 mm resolution) with a multi-band factor of 5 echoplanar-imaging (EPI) sequence (repetition time [TR] = 2000 ms, acquisition time [TA] = 1100 ms, field of view [FOV] = 210 mm × 210 mm, echo time [TE] = 35.8 ms). Each volume consisted of 50 slices (no gap), covering the whole brain. The structural data (1 mm × 1 mm × 1 mm) was acquired using a T1-weighted three-dimensional MPRAGE sequence ([TR] = 2300 ms, [TE] = 2.98 ms, 192 sagittal slices).

Area of acquisition

Whole brain

Diffusion MRI ☐ Used ☒ Not used

Preprocessing

Preprocessing software

BrainVoyager QX version 2.8 and BrainVoyager versions 20.6 and 21.4 (Brain Innovation, Maastricht, The Netherlands). Standard pre-processing was performed. Anatomical data underwent inhomogeneity correction, Talairach transformation and segmentation (with manual adjustments) to create individual cortical surface representations per hemisphere. These were then combined into a group-averaged surface representation using cortex-based alignment. To ensure all anatomical runs were well aligned across sessions, the native space anatomical data of sessions 2 and 3 was coregistered to the ACPC transformed session 1 anatomy using vmr-vmr coregistration in Brainvoyager QX. The resulting transformation file was subsequently applied to the anatomical runs of sessions 2 and 3 resulting in well aligned anatomical data. Transformation to Talairach space was performed using the transformation file of session 1. Functional data underwent slice scan time and motion correction, high-pass temporal filtering and were subsequently aligned across sessions by co-registration to the Talairach transformed anatomical data of session 1, re-sampled to 3 mm iso-voxel resolution and spatially smoothed using a 6 mm FWHM Gaussian kernel.

Normalization

Coregistration to Talairach space.

Normalization template

Talairach

Noise and artifact removal

Volumes of functional runs affected by excessive head motion (≥ 3 mm/degree translation/rotation in any direction) were removed from the run. If the number of affected volumes exceeded 20%, the run was excluded from further analyses.

Volume censoring

Volumes described above were cropped from the affected runs using custom MATLAB routines.

Statistical modeling & inference

Model type and settings

Random effects general linear model followed by RM ANOVA to explore session effects.

Effect(s) tested

Cortical activation for each session was assessed using contrasts (t-statistics) comparing activation in response to the audio-visual exposure blocks compared to fixation cross baseline. Session effects were assessed using RM ANOVA with exposure blocks of the 3 measurement sessions as the within-subject variable. This resulted in an F-statistic map of brain areas that show a main effect of session.

Specify type of analysis: ☒ Whole brain ☐ ROI-based ☐ Both

Statistic type for inference
(See Eklund et al. 2016)

Whole-brain FDR threshold of q<0.05

Correction

FDR

Models & analysis

n/a	Involved in the study
☒	☐ Functional and/or effective connectivity
☒	☐ Graph analysis
☒	☐ Multivariate modeling or predictive analysis
